# Diabetes during Pregnancy: A Transgenerational Challenge

**DOI:** 10.3390/jcm12062144

**Published:** 2023-03-09

**Authors:** Stefano R. Giannubilo, Andrea Ciavattini

**Affiliations:** Department of Clinical Sciences, Polytechnic University of Marche, Salesi Hospital, Via Corridoni 11, 60123 Ancona, Italy

For many years, gestational diabetes mellitus (GDM) has been defined as “a glucose intolerance of variable magnitude that begins or is first diagnosed in pregnancy” and that, in most cases, resolves after delivery [1]. This definition was also used in the past for those forms of pre-gestational diabetes that were first identified in pregnancy. The significant increase in the prevalence of type 2 diabetes (DT2), even in childbearing age, has made it common to find women with diabetes, usually type 2, undiagnosed who undertake pregnancy. These women have an increased risk of maternal and fetal complications, including congenital malformations [2]. This condition, termed “overt diabetes in pregnancy”, requires a pregnancy follow-up similar to that recommended in pre-gestational diabetes. In recent years, major guidelines have recommended that specific attention be given to screening for overt diabetes in pregnancy, which should be performed as early as possible, in the same manner as recommended outside pregnancy. GDM, on the other hand, usually arises in the second part of pregnancy, which is why the optimal time for screening is 24–28 weeks of gestation [3]. Some particularly high-risk conditions, such as obesity, prior GDM, and impaired fasting blood glucose before or early in pregnancy may result in the early onset of GDM. Although strong scientific evidence is not available, an approach that takes lifestyle modifications and early screening at 16–18 weeks of gestation, to be repeated, if negative, at 24–28 weeks is recommended. Two large randomized clinical trials [4,5] have clearly demonstrated that treatment of GDM reduces the incidence of adverse pregnancy outcomes, even in forms with mild blood glucose changes. The diagnosis of GDM is therefore relevant to pregnancy outcome and also represents an important opportunity for the prevention of diabetes in the mother [6]. Since the oral glucose-loading curve (OGTT) [7] was first introduced, scientific debate over the years has focused on the type of test, the best gestational age, and the population to be tested (universal or selective screening), and to date, there is still no precise guidance. A slight majority of relevant observational studies report an improved pregnancy outcome by treatment of early-onset GDM. However, so far, RCTs have not provided conclusive evidence of the beneficial effects of early treatment and regard the appropriate early-pregnancy OGTT thresholds for the diagnosis of GDM and for the assessment of the impact of early treatment on obstetrical outcomes and long-term offspring health [8]. The major shortcoming of a test that detects only blood glucose lies in the fact that the fetal consequences of diabetes in pregnancy involve a number of placental, endocrine, and metabolic factors that are not exhausted by the simple assessment of maternal blood glucose. In addition, the OGTT, which as proposed today is performed at 16–18 weeks at the earliest, would not cover the possible consequences of maternal and/or fetal hyperglycemia in the early pregnancy. Several alternatives to OGTT have been suggested. The use of HbA1c for diagnosing GDM has been disappointing because there is substantial overlap between women with normoglycemia and women with GDM [9]. Some other strategies include a single random glucose measurement; however, this demonstrated low sensitivity [10]. A single random fasting glucose has been shown to reduce the rate of women undergoing OGTTs by more than 80%, while it identifies around 70% of the women with GDM, especially those with the highest risk of adverse outcomes [11]. The real problem is that hyperglycemia, or hyperglycemia stimulated by glucose loading, is only one, perhaps the most obvious, of the aspects of diabetes in pregnancy. The best paradigm that diabetes finds its basis in placental endocrine and inflammatory biology is the association with hypertensive disorders of pregnancy (HDP) and fetal growth defects. Maternal insulin resistance has been implicated in the pathogenesis of HDP due to the existence of marked hyperinsulinemia during pregnancy before the development of HDP [12]. Regarding the pathophysiological overlap between diabetes and hypertension in pregnancy, it has been shown that metformin reduces the risk of HDP in obese patients with GDM [13]. Additionally, a higher sFlt-1/PIGF ratio (two angiogenetic factors) contributed to the increased incidence of HDP and obstetric as well as perinatal complications in women with GDM [14]. In addition to angiogenetic factors, research is exploring numerous biomarkers such as cytokines (Adipokines, Chemerin, Fetuin, Leptin, Omentin, Interleukin 6), glycoproteins (Afamin, CD59), and other proteins (Nesfatin-1, PAPP-A, RBP4) [15] with the goal of better exploring early in pregnancy the complex universe of endocrine changes to which a fetus is subjected during the diabetes of pregnancy and which program infants who will more easily become obese, hypertensive, diabetic adults with metabolic syndrome. An abundance of evidence now shows that increased maternal blood glucose results in an increased risk of fetal and newborn morbidity [16,17], and this is probably the consequence of fetal hyperinsulinemia induced by excess maternal glucose. In fact, the transplacental glucose passage results in fetal hyperglycemia, compensatory hyperinsulinemia resulting in excessive development of insulin-sensitive tissues (adipose tissue, skeletal and myocardial muscle, liver, islets of Langerhans), accelerated fetal growth and thus macrosomia, and, after umbilical cord resection, neonatal hypoglycemia. The Hyperglycaemia and Adverse Pregnancy Outcome (HAPO) study [18] identified a strong association between maternal glycemia and infant anthropometry-derived adiposity and adiposity in childhood is associated with type 2 diabetes and cardiovascular disease in adult life [19]. In particular, excess fuels in the gestational environment may lead to increased hepatic fat deposition in the fetus, which possibly plays a role in the development of nonalcoholic liver disease in children [20]. The phenotypes of the infants exposed to treated GDM have an increased intra-abdominal adiposity that is associated with several metabolic disorders [21]. In addition, in the umbilical cord tissue, the methylation status of the DNA in the promoter of the gene encoding for the nuclear receptor of retinoids (RXRA) is predictive of the degree of adiposity in pediatric age and the risk of type 2 diabetes [22]. These data support the hypothesis that the identification of epigenotypes at birth may have prognostic value and prove useful for monitoring the outcomes of intervention strategies aimed at optimizing health status and maternal nutrition, with long-term benefits for the offspring. The treatment of diabetes in pregnancy turns out to be a duty to the mother, to the child, and, from an epigenetic perspective, to future generations. This evidence has fascinated many scientists, who believe that the dramatic transformation of the environment wrought by humans over the course of a few decades and the spread of artificial molecules, which are capable of interfering with epigenetic mechanisms of programming fetal, are at the origin of the “earthquake epidemiological” responsible for the pandemic of obesity and DT2, or more generally, of the rapid increase in and manifestation of numerous chronic-degenerative diseases at an early age. This is the major theme elaborated in the so-called Barker Hypothesis [23] and, more recently, in the Fetal Origin Theory of Adult Disease [24]. The epigenome constitutes the interface between genes and the environment, by virtue of the plasticity of epigenetic traits and their susceptibility to environmental influences. It represents the specific “place” where the flow of information from the environment is integrated with that contained in the DNA sequence, so as to coordinate the molecular processes that determine the structure and function of cells and tissues, contributing to the transformation of the phenotype, both physiological and pathological ones. The epigenome can, therefore, be considered a molecular “recorder” of the environmental exposures, which follow one another and accumulate over the course of each individual’s existence. The novelty of the message contained in Barker’s Hypothesis, is that pre-natal life is not fully protected in the uterine microenvironment. The exposure of the mother to certain environmental factors can alter the fetal epigenome, interfere with the normal process of cell differentiation, and alter the programming of tissues and organs in ways that are not infrequently irreversible and sometimes even transmissible from one generation to the next [25]. This is defined as transgenerational epigenetic inheritance of the transmission through the germline of a given epigenetic set-up in the absence of direct exposure of the subject to (epi)genotoxic agents [26]. From this perspective, several studies have shown that exposure in utero to maternal glucose dysmetabolism or gestational diabetes alters, in the umbilical cord, the DNA methylation of genes involved in metabolic energy, such as LEP and ADIPOQ, and in growth, such as IGF1R and IGFBP3, and of imprinted genes, such as MEST [27]. Since the alteration of the expression of these genes contributes to the development of metabolic diseases in adults, the epigenotypes identified could join the list of potential biomarkers epigenetics of adverse postnatal outcomes. The process that leads a candidate biomarker to become a tool that can be used in clinical practice is complex and must be considered from a long-term perspective. Indeed, prospective studies of a long duration and large case series demonstrate the ability of the biomarker to independently predict the development/progression of the disease. In addition, studies need to be conducted on the intervention to verify that it is capable of providing prognostic information and to guide intervention therapy. What appears to be more promising is the use of different circulating miRNAs as biomarkers for diabetes [28]. The increased prevalence of GDM in all nations of the world has emphasized the importance of prevention. An increasing number of women begin pregnancy in an obese condition or at an advanced age contributing to the significantly increasing trend of GDM. Interventions effective in preventing type 2 diabetes could be effective in preventing GDM. A meta-analysis of randomized clinical trials [29] identified three different preventive strategies: diet, diet plus lifestyle, and nutritional supplementation. The GDM recurrence rate is high, with approximately 50% of women experiencing this same diagnosis in their subsequent pregnancy. Examination of large datasets suggests that the risk factors for GDM recurrence are similar to those for GDM itself and include increasing maternal age, weight and certain ethnicities. In addition, women who require insulin to treat GDM in the index pregnancy seem more likely to experience recurrence [30].

In conclusion, the prevention of diabetes in pregnancy with proper lifestyle, early diagnosis, and the study of new biomarkers of diabetes in pregnancy are now a duty of the scientific community to future generations.
